# Harnessing the power of midwifery: Transforming self-efficacy and health navigation among women with polycystic ovarian syndrome

**DOI:** 10.6026/973206300212724

**Published:** 2025-08-31

**Authors:** Jessy Raja, Sumathi Chandran, Shankar Shanmugam Rajendran, Vanitha Narayanasamy Naidu, Amudha Chinnappan, Azhgar Jan Thoulath Khan, Atchaya Rajagopal

**Affiliations:** 1 Department of Obstetrics and Gynecological Nursing, College of Nursing, Madras Medical College, The Tamil Nadu Dr. MGR Medical University, Chennai, Tamil Nadu, India; 2 Department of Obstetrics and Gynaecology, Institute of Obstetrics and Gynecology, Government Hospital for Women and Children, Madras Medical College, The Tamil Nadu Dr. MGR Medical University, Chennai, Tamil Nadu, India; 3 Department of Child Health Nursing, College of Nursing, Madras Medical College, The Tamil Nadu Dr. MGR Medical University, Chennai, Tamil Nadu, India; 4 Department of ommunity Health Nursing, College of Nursing, Madras Medical College, The Tamil Nadu Dr. MGR Medical University, Chennai, Tamil Nadu, India

**Keywords:** PCOS, self-efficacy, health information-seeking behaviour, midwife-led intervention, women's health, quasi-experimental study, reproductive care

## Abstract

The impact of a midwife-led intervention on self-efficacy and health information-seeking behavior (HISB) in women with Polycystic Ovarian Syndrome (PCOS) is of interest. Sixty women were divided into an experimental and a control group, with the experimental group receiving a structured intervention over four weeks. Results showed significant improvements in self-efficacy and HISB in the experimental group, compared to no change in the control group. Thus, we show that midwife-led interventions effectively empower women by enhancing their ability to manage PCOS. It emphasizes the importance of integrating such interventions into routine gynecological care.

## Background:

A woman is a symphony of strength, resilience, grace, and quiet power. From the miracle of giving birth to the silent sacrifices she makes in the background, her journey is marked by battles both visible and unseen. Yet behind her composed smile, she often hides unspoken struggles of hormones that misbehave, of pain that's misunderstood, of cycles that betray her dreams of motherhood [1]. Polycystic Ovarian Syndrome was first described in 1935 by Irving Stein and Michael Leventhal, who noted a combination of amenorrhea, obesity and hirsutism in affected women [2]. It is also called Stein-Leventhal Syndrome or Hyperandrogenic Anovulation” and is the most common endocrine disorder affecting approximately 6-26% women of Reproductive age. Polycystic Ovarian Syndrome is characterised by the presence of numerous small, underdeveloped follicles in the ovaries, usually less than 8mm in size. These follicles fail to release mature eggs, resulting in anovulation [3]. We are living in a period of modernization. The effect of modernization and technological advancement is reflected in everyday life, unhealthy food habits and lack of exercise lead to many diseases in reproductive age women, especially polycystic ovarian syndrome [4]. Polycystic Ovarian syndrome requires "control" rather than "cure" and the focus of treatment is on seeking treatment. Health Information Seeking Behaviour refers to the proactive effort individuals make to find reliable health-related information to understand and manage their condition [5]. Self-efficacy refers to the belief in one's ability to perform the actions necessary to achieve specific health goals. Strong self-efficacy is associated with positive health behaviors, enhanced well-being, and improved adherence to treatment regimens [6]. Women with Polycystic Ovarian Syndrome often struggle with low self-efficacy due to body image issues and irregular menstruation [7]. Midwives are now recognised as essential figures in women's health, not only in childbirth but across all life stages. In the context of polycystic ovarian syndrome, midwives are in an ideal position to deliver holistic, woman-centred care through interventions that include counselling, lifestyle modification support, nutritional guidance, and emotional empowerment [8]. Therefore, it is of interest to transform self-efficacy and health navigation in women with polycystic ovarian syndrome.

## Methodology:

The study employed a quasi-experimental pre-test and post-test control group design, with 30 women with PCOS in each of the experimental and control groups. Non-probability purposive sampling was used. The study was conducted in the Gynaecology Department at the Institute of Obstetrics and Gynaecology (IOG), Chennai, over four weeks. The tool consisted of three sections: demographic variables, a health information-seeking behavior questionnaire (25 items; scores 25-125), and a self-efficacy scale (10 items; scores 10-40). Content validity was established by experts, and reliability was confirmed using the test-retest method. Data were collected through structured interviews, which took 25-30 minutes per participant, and were analyzed using both descriptive and inferential statistics. Ethical clearance was obtained from the ethical committee and the Director of the Institute of Obstetrics and Gynaecology (IOG). The ethical principles followed included beneficence, respect for dignity, confidentiality, and informed consent, ensuring the rights and privacy of participants.

## Results:

The study revealed that in the pre-test, 70% of the experimental group and 65% of the control group had low self-efficacy, while 86.7% and 90%, respectively, had low HISB. Post-intervention, 86.7% of the experimental group showed high self-efficacy, and 93.3% demonstrated high HISB scores, compared to 0% in the control group. The mean self-efficacy score in the experimental group increased from 19.34 ± 2.84 to 31.32 ± 3.57 (p < 0.001), while the HISB score rose from 58.1 ±5.4 to 122.5 ±7.8 (p < 0.001). Significant associations were observed between post-test self-efficacy and variables such as family income (p = 0.023) and type of family (p = 0.004), as well as between HISB and variables like BMI (p < 0.05) and type of treatment (p < 0.05). Table 1 (see PDF) displays the comparison of pre-test and post-test levels of self-efficacy in the experimental and control groups. The experimental group showed a significant increase in self-efficacy from a pre-test mean of 19.34 ± 2.84 to a post-test mean of 31.32 ± 3.57 (t = 16.44, p < 0.001), while the control group did not show a significant change, with pre-test and post-test means of 20.02 ± 2.56 and 20.02 ± 2.56, respectively (t = 1.89, p > 0.05). Table 2 (see PDF) illustrates the comparison of pre-test and post-test levels of health information-seeking behavior in the experimental and control groups. The experimental group exhibited a substantial improvement, with the pre-test mean of 58.1 ± 5.4 increasing to a post-test mean of 122.5 ± 7.8, yielding a mean difference of 64.4 (t = 29.18, p < 0.001). In contrast, the control group had a minimal change, with a pre-test mean of 60.2 ± 6.0 and a post-test mean of 62.1 ± 6.5, resulting in a mean difference of 1.9 (t = 1.57, p > 0.05). Figure 1 (see PDF) shows the comparison of pre-test and post-test levels of self-efficacy in the experimental and control groups. Figure 2 (see PDF) presents the comparison of pre-test and post-test levels of health information-seeking behavior in the experimental and control groups.

## Discussion:

The comparison of pre-test and post-test self-efficacy scores between the experimental and control groups showed that in the experimental group, the mean self-efficacy score increased significantly from 19.34 ± 2.84 in the pre-test to 31.32 ± 3.57 in the post-test. The computed t-value was 16.44 with a p-value of < 0.001, indicating a highly statistically significant difference. This suggests that the midwife-led intervention was highly effective in enhancing the self-efficacy of women with polycystic ovarian syndrome. Ariani *et al.* (2022) [9] conducted a pre-experimental one-group pretest-posttest study among 261 senior high school girls in Denpasar, Bali, to assess the influence of video-based health education on early screening efforts for Polycystic Ovary Syndrome (PCOS). Using a structured questionnaire and the Wilcoxon Rank Test, the study found significant improvements in knowledge (p = 0.001), attitude (p < 0.001), and behavior (p < 0.001) following the intervention. The results highlight the effectiveness of video-based learning in enhancing awareness and proactive behavior toward PCOS early screening among young women. Williams (2017) [10] evaluated the effectiveness of a community-based intervention package in preventing Polycystic Ovarian Syndrome among Adolescent girls residing in Choolai. The study utilized a one-group pre-test and post-test design. The results demonstrated that the community-based intervention package showed a significantly higher improvement in knowledge regarding the prevention of polycystic ovary syndrome, as determined by a paired t-test, p < 0.05. These findings indicate that the intervention is a more effective strategy with essential knowledge among adolescent girls and positive attitudes toward managing and understanding PCOS.

## Conclusion:

A midwife-led intervention significantly improves health information-seeking behaviour and self-efficacy among women with PCOS. Thus, we show the critical role of midwives in reproductive health education and the need for integrating such structured, nurse-led interventions into routine gynecological care to empower women in managing chronic reproductive disorders.
